# Effects of antidepressant treatment on heart rate variability in major depression: A quantitative review

**DOI:** 10.1186/1751-0759-2-12

**Published:** 2008-06-30

**Authors:** Louis T van Zyl, Takuya Hasegawa, Katsutaro Nagata

**Affiliations:** 1 Department of Psychiatry, Division of Psychosomatic Medicine & Consultation-Liaison Psychiatry, Queen's University at Kingston General Hospital, Connell-4, Suite 2-486, Kingston, ON K7L 2V7, Canada; 2 Hamamatsu University, School of Medicine, Japan, Hamamatsu University School of Medicine, 1-20-1, Handayama, Hamamatsu City, 431-3192, Japan

## Abstract

**Background:**

The literature measuring effects of antidepressant and electroconvulsive therapy (ECT) for major depression on heart rate variability (HRV) in medically well individuals was reviewed.

**Methods:**

Fourteen studies evaluating HRV were included. Twenty three pre-post or within group comparisons were available. Treatment impact on measures of HRV was pooled over studies. We examined different classes of antidepressants, and for short and long electrocardiogram (ECG) recordings separately.

**Results:**

Tricyclic antidepressants (TCAs) were associated with declines in most measures of HRV and significant increase in heart rate (HR) in studies with short recording intervals. No significant changes were found for longer recording times.

Treatment effects with selective serotonin reuptake inhibitors (SSRIs) were more variable. Short-recording studies revealed a significant decrease in HR and an increase in one HRV measure. In two 24-hour recording studies no significant changes were observed. No relationship between ECT and HRV has been established in the literature. The effects of other drugs are reported.

**Limitations:**

Few studies measure the effects of treatment of depression on HRV. Existing studies have generally used very small samples, employing a variety of measurements and methodologies.

**Conclusion:**

We confirm that TCAs are associated with a large decrease in HRV and increase HR. However, data for SSRIs is not clear. Although the effect of SSRIs on HRV is weaker than for TCAs, evidence shows that SSRIs are associated with a small decrease in HR, and an increase in one measure of HRV. The use of TCAs in depression leads to changes in HRV that are associated with increased risk of mortality.

## Background

Heart rate variability (HRV) refers to the beat-to-beat alterations in heart rate. Its measurement is used to assess cardiac autonomic function and it is related to outcomes following cardiac events [1-5]. There is some evidence that major depressive disorder (MDD) is associated with decreased heart rate variability [6-9], and that medications used in the treatment of MDD also affect HRV. HRV in depression and in its treatment is an important issue since both depression and decreased HRV have been shown to be predictors of mortality in cardiac patients. Moreover studies have shown that depression is associated with a higher rate of development of coronary heart disease and of death after an index myocardial infarction (MI) [10], and that a low HRV after MI is associated with considerable risk of mortality [2,11-14] As a result there has been substantial interest in the relationship of HRV with depression and with antidepressant medications.

The purpose of the current paper is to provide an overview of the literature examining the effects of pharmacologic and physical treatments for depression on HRV in medically otherwise well patients with MDD. The present paper describes the methodologies and HRV measures used in the study of the effects of treatment of depression on HRV, and reviews the effects of two major classes of antidepressants on HRV: tricyclic antidepressants (TCAs) and selective serotonin reuptake inhibitors (SSRIs). In addition, we review the small group of studies examining the effects of other antidepressants on HRV, as well as the effects of electroconvulsive therapy (ECT).

### Defining and Measuring Heart Rate Variability

Before reviewing the relationship between HRV and treatments for depression, it is important to first describe methods for measuring HRV. In studies examining the effects of treatments for depression on HRV a variety of measures of HRV have been employed across different studies, and as a result, there is a lack of consistency in approach to HRV measurement. Four common approaches to measurement of HRV (beat-to-beat alterations in heart rate) are: time domain, spectral or frequency domain, nonlinear, and geometric methods [15,16].

Because time- domain and spectral or frequency domain methods are the two methods generally used for assessing the effects of psychiatric treatments for depression on HRV, we briefly describe these below.

### HRV Measures: Time Domain Methods

Time domain methods reflect various statistical approaches of measuring and representing the differences in the intervals between adjacent normal R waves, referred to as normal to normal (NN) intervals, over a specified period of time [15]. The Task Force of the European Society of Cardiology (1996) describes seven time domain methods. Four of these have been reported in the literature on HRV and antidepressants. These include SDNN, (the standard deviation of all NN intervals); SDANN, (the standard deviation of means of all NN intervals measured from successive five-minute recording segments over a 24-hour assessment time) RMSSD, (the square root of the mean of the sum of squares of differences between all adjacent NN intervals), and pNN50, (the percentage of NN interval differences >50 ms from the preceding interval).

Of the four time domain methods, SDNN is the simplest to compute and the most commonly used measure of HRV.

The different time domain measures are thought to be influenced by different factors. Both SDNN and SDANN are thought to reflect changes of HRV mediated by both the influence of parasympathetic efferent and sympathetic efferent nerve factors. RMSSD and pNN50 are thought to reflect the changes of HRV mainly mediated by parasympathetic efferent nerves [15]. Bigger et al have reported associations of decreases in time domain measures with increased cardiac mortality [17,18]. After adjusting for other risk factors, SDANN emerged as the most powerful predictor of cardiac mortality, followed by pNN50.

RMSSD and SDNN were less powerful but were still significant predictors of mortality [18]. Because of the moderate correlation between heart rate and HRV the coefficient of variation (CV) calculation is sometimes also reported in which SDNN in each five minute interval is divided by the mean NN interval (SDNN/NN) which attempts to control for differences in heart rate [19].

### HRV Measures: Frequency-domain Methods

Time domain methods focus on variations in the length of the interbeat interval. Although spectral domain methods also take this into account by breaking it down into specific components at each frequency, the focus is on frequency variations. Frequency domain HRV measures the underlying variance (power) in the heart rate pattern at different frequencies. Short-term (two to five minute) electrocardiogram (ECG) recordings for spectral analysis tend to produce three peaks, a high frequency (HF) peak between 0.15 and 0.40 Hz, a low frequency (LF) peak between 0.04 and 0.15 Hz, and a very low frequency (VLF) peak below 0.04 Hz [20]. Twenty-four hour ECG recordings also provide an ultra-low frequency (ULF) peak below 0.003 Hz [11,21]. Total power represents the total variability in the record.

HF power reflects HRV attributable to respiratory sinus arrhythmia, and is regarded as a marker of vagal modulation of R-R intervals and a cardiac parasympathetic effect [22]. In twenty-four hour recordings, HF power correlates most highly with RMSSD and pNN50. LF power may be modulated by baroreflex activity, and appears to reflect sympathetic and parasympathetic influence on the RR intervals [23,24]. In twenty-four hour recordings, LF power correlates most highly with SDNN averaged over five minute intervals. It is argued that the LF/HF ratio estimates sympathovagal balance However, the measurement of sympathovagal balance has been the subject of controversy [25,26].

VLF power represents a measure of uncertain value. VLF power may indicate thermoregulation or vasomotor activity, although this has been disputed [27,28]. It may involve a parasympathetic component [28,29] and possibly engage the rennin-aldosterone system [28,30,31]. ULF power is far less clear, but has a strong relationship with SDANN [11].

### HRV Measures – Spectral Methods

Non-linear measures of HRV have been demonstrated to be potentially superior in predicting sudden death in post-infarction patient populations [32,33]. Yeragani et al studied the effects of paroxetine and nortriptyline on HRV in patients with ischemic heart disease and major depression employing various methods of HRV measuring, including non-linear components of HRV. Evidence suggests that non-linear assessments may provide additional information about cardiac autonomic modulation, and more particularly about vagal modulation [34,35]. However, these measures have not been reported in studies of the effects of antidepressants on healthy volunteers.

### Factors Affecting HRV

Various factors influence the accuracy and usefulness of the HRV components being measured. These include the duration of the recording and the effect of the various physical and physiological changes that occur during the recording period [15,36].

Spectral analysis of short-term, two to forty minute ECG recordings provides information about the autonomic status of the heart, reflecting mainly vagal activity [15]. Short recording times hold the advantage over long recording times only in as far as the shorter duration makes it possible to create physiologically fixed or stationary states, without too much difficulty; whereas with long-term recordings, it is harder to create and maintain strict standardized conditions. Longer recording times are needed for measurement of low frequency (VLF and ULF) components of the recoding and LF and HF components are best suited to short-term recording times [15,37].

In addition to difficulties with maintaining a steady state, long-term recording times also pose difficulties with antidepressant studies in particular. The overall effect of the natural environment on the autonomic nervous system and its influence on the heart is best assessed over a longer 24-hour recording period. However, they are problematic for drug studies because the antidepressants themselves can cause both physiological changes and behavioural changes (e.g. increased activity) both of which may influence a twenty-four hour recording. For this reason it seems that with drug studies, short recordings under standardized conditions (e.g. supine) may give a purer view of physiological changes.

Both short and long recordings have shown a relationship between decreased HRV and mortality. Almost all of the early predictive studies of mortality used twenty-four hour recordings but recent studies have shown a similar relationship using shorter recordings [18].

Long-term and short-term recordings each have their advantages and their disadvantages [36]. It is likely that this fact that has led to the variation in approaches to the assessment of HRV changes associated with antidepressants. In addition, in research examining the effects of antidepressants on cardiac autonomic function there is no clear consensus as to which HRV measures are most valuable to assess, nor which would be the preferred recording-time duration (i.e., short or long), and there is rarely a justification given for the choice of measures in these investigations.

The present study reviews the evidence describing changes in HRV associated with pharmacological and physical treatments for depression. Different measures of HRV are examined separately as are measures from short and long recordings. Classes of drugs (TCAs and SSRIs) are also examined separately.

## Methods

Searches were made in Medline and PsycInfo for studies including the terms "antidepressant" (and specific antidepressant drugs) combined with "heart rate variability" or "heart period variability", or "RR variability". A total of 65 potential papers resulted from these searches, in addition to potential papers referenced in these papers. Studies were selected for the present study based on the following criteria. First, patients were adults (aged 18 or more) with major depression as assessed by the Diagnostic and Statistical Manual of Mental Disorders (DSM) criteria appropriate for the year of the study. Second, patients were administered an antidepressant, or ECT. Third, HRV was assessed and could be compared either pre- or post-drug initiation, or between a treated group and an untreated or placebo contrast group. Fourth, if a study included two treatment conditions it was only included if the participants were randomly assigned to treatments. Fifth, studies using a variety of drugs were only included if the data were broken down by each specific drug. In cases where comparisons between a treatment group and a control group within the treatment group (pre-post) were possible, only the pre-post data were included since this was the only comparison in the majority of the studies. Fourteen papers met these criteria, and since some of these examined more than one treatment a total of 23 comparisons were possible.

Effect sizes (Cohen's *d*) were computed for each comparison. In instances where standard deviations for a particular study were not reported they were estimated as the mean of the standard deviations reported in the other studies in the group. Calculation of *d *in repeated measures requires an estimate of the correlation between the measures at the two observations. In cases where this could not be estimated from the data reported, the median of test-re-test correlations for the particular HRV measure found in the literature [19,38-48] was employed.

The variables summarized were heart rate, and time domain measures (RMSSD, SDNN, SDANN, pNN50, and CV), as well as frequency domain measures of HRV (HF, LF, lnHF, and LF/HF). These values were included because they were the measures most often reported in the studies examined. (The log transformation of HF, lnHF, was examined separately from HF). Studies reported one or several measures of HRV and there was considerable diversity in the measures reported and the length of the recording time. In some instances missing values were calculated from the available data if they were not specifically reported in the paper (e.g. CV from SDNN and HR in five minute recordings). Data for frequency domain variables were included only if the frequency ranges reported corresponded closely to the ranges set out by the Task Force as some early studies used different ranges [49].

Values were grouped by length of ECG recording (short versus 24 hour). In short recording studies, only data from the supine condition were included, as this was the condition common to most studies. Almost all short recording time studies used five-minute recordings. (range = 2 to 8 minutes). The statistical significance of the difference of mean values of *d *from zero was calculated using one sample *t *tests or between group *t *tests as appropriate.

This paper is a review of the literature that did not require research ethics board approval.

## Results

### 1. HRV Response in Patients with MDD treated with TCAs

The only TCAs studied were amitriptyline, imipramine and doxepin. Data from the studies yielded 10 comparisons for the effects of specific TCAs on HRV. All but two of these comparisons used short recording times. The summary results are shown in Table 1, and the data for each study is presented in Table 3.

**Table 1:** Patients with MDD using TCA and SSRI medication where Cohen's *d *reflects the difference between treatment and contrast condition.

**Measure**	**Heart Rate**	**SDNN**	**SDANN**	**RMSSD**	**LF**	**LnHF**	**HF**	**LF/HF**	**CV**	**pNN50**
**Units**	**bpm**	**ms**	**ms**	**ms^2^**	**ms^2^**	**ms^2^**	**ms^2^**	**ratio**	**ratio**	**percent**
TCAs – short (2–10 minute) supine										
Mean Baseline Value	75.91	32.28		21.60		5.80	0.51		4.07	
Mean Percent Change	20.8%	-62.9%		-67.3%		-35.5%	-79.4%		-55.2%	
Number of Comparisons	8	8		6		2	4		8	
Mean Effect Size	4.83	-2.43		-3.34		-4.54	-0.96		-5.52	
*t*	7.53*	9.32*		7.79*		2.40	5.85*		4.17*	
*p*	0.00	0.00		0.00		0.25	0.01		0.00	
**TCAs 24 hour ambulatory**										
Mean Baseline Value	82.60	115.85	106.7	31.00						10.00
Mean Percent Change	7.5%	-16.1%	-3.7%	-12.9%						-30.0%
Number of Comparisons	1	2	2	1						1
Mean Effect Size	1.98	-0.20	0.31	-2.71						-3.34
*t*		0.10	0.10							
*p*		0.94	0.94							
**SSRIs – short (2–10 minute) supine**										
Mean Baseline Value	76.00	28.67	26.375	26.38		5.60	0.507		3.60	
Mean Percent Change	-2.0%	1.0%		-2.4%		-1.4%	-14.8%		-1.9%	
Number of Comparisons	4	3		4		1	1		3	
Mean Effect Size	-0.63	0.06		1.42		-0.15	-0.26		-0.26	
*t*	6.33*	3.46**		0.54					2.71	
*p*	0.01	0.07		0.63					0.11	
**SSRIs 24 hour ambulatory**										
Mean Baseline Value	81.30	30.08	118.1	30.00						9.00
Mean Percent Change	-0.5%	1.7%	0.0%	16.7%						33.3%
Number of Comparisons	1	2	2	1						1
Mean Effect Size	-0.10	0.51	0.43	1.76						1.76
*t*		0.30	0.41							
*p*		0.82	0.75							

Two studies used 24 hour monitoring. Only studies have been included where effects of specific drugs could be evaluated. Negative values indicate decreases in the parameter with treatment, positive values indicate increases. (* indicates *p *< .05, ** indicates *p *< .10)

In the short recording studies (where patients were supine), treatment with the TCAs led to a sharp decrease in all measures of HRV reported, and to an increase in heart rate. The mean effect sizes for all measures were very large ranging from 2.40 to 9.32 indicating a powerful effect. However, in the two long recording studies (where patients were ambulatory), the changes were smaller and inconsistent. Lederbogen's [50] results showed decreases in SDNN and SDANN, whereas Khaykin [51] found opposite results, with significant *increases *in SDNN and SDANN, as well as a decrease in RMSSD. Generally, the findings in the short recording studies were strong and robust, demonstrating greatly decreased HRV on a number of measures, while the findings in the longer recording studies were weaker and markedly inconsistent.

### 2. HRV Response in Patients with MDD Treated with SSRIs

Papers investigating HRV response to SSRI treatment yielded a total of seven comparisons. The medications investigated in these studies were fluvoxamine, paroxetine, and fluoxetine. In five of the comparisons, HRV parameters were obtained from short recordings and in two studies 24-hour recording methods were employed (See Table 1).

With respect to the short-term recording studies there was a significant decrease in HR associated with the drug treatment in all five studies. The change in HR was much smaller and in a different direction than that in the TCA studies. The only reliable change in HRV was a marginally significant increase in SDNN. The very wide range of values for the different parameters (see Table 3) and the small number of studies limits the power of this analysis. As in the TCA comparisons, the results of the two long-term (24-hour) recording studies were markedly contradictory. Lederbogen [50] found little change in HR, but again found moderate and significant decreases in SDNN and SDANN. Conversely, Khaykin [51] found moderate increases in SDNN and SDANN, as well as an increase in RMSSD. In summary, the short-term recordings of patients treated with SSRIs showed a decrease in HR, and a marginally significant increase only in SDNN. Again, the long-term studies were markedly contradictory, and thus it is difficult to comment on these results.

### 3. HRV Responses in Patients with MDD with Other Treatments

Table 2 sets out the results of studies examining other pharmacological and physical treatments of depression. The treatments are diverse and include ECT, bupropion, mirtazapine, nefazodone and reboxetine. The significant changes (as reported by the authors or in our re-analysis of the data from one study [52] were confined to an increase in HR with nefazodone and with mirtazapine, and decreases in all reported measures of HRV with bupropion and mirtazapine. No significant effects were found for ECT or reboxetine. No attempt was made to combine the effect sizes across studies because of differences in classes of specific drug or differences in recording time.

**Table 2:** Changes in HRV related to physical treatments for depression other than SSRIs or TCAs.

**Measure**	**Heart Rate**	**SDNN**	**SDANN**	**RMSSD**	**LF**	**lnHF**	**HF**	**LF/HF**	**CV**	**pNN50**
**Units**	**bpm**	**ms**	**ms**	**ms**	**ms^2^**	**ms^2^**	**ms^2^**	**ratio**	**ratio**	**percent**
**Reboxetine short supine**										
Mean Baseline Value	82.60			18.50	946.00		354			
Mean Percent Change	5.6%			-1.6%	-18.9%		21.8%			
Number of Comparisons	1			1	1		1			
Mean Effect Size	1.32 ns			-0.04 ns	-0.38 ns		0.79 ns			
**Nefazodone short supine**										
Mean Baseline Value	75.80	30.08		24.50	864.00		480	2.97	3.80	
Mean Percent Change	-5.9%	14.3%		4.1%	-5.7%		-10.4%	-5.7%	-2.6%	
Number of Comparisons	1	1		1	1		1	1	1	
Mean Effect Size	-1.60 *	0.08 ns		0.11 ns	-0.08 ns		-0.20 ns	-0.16 ns	-0.21 ns	
**Bupropion short supine**										
Mean Baseline Value				28.10						
Mean Percent Change				-42.7%						
Number of Comparisons				1						
Mean Effect Size				-1.99 *						
**Mirtazapine short supine**										
Mean Baseline Value	66.00	49.60				6.50			5.40	
Mean Percent Change	15.29%	-35.7%				-21.5%			-25.9%	
Number of Comparisons	1	1				1			1	
Mean Effect Size	3.10 *	-2.47 *				-1.81 *			-3.97 *	
**ECT short supine**										
Mean Baseline Value					548.00		264.00			
Mean Percent Change					-42.9%		50.4%			
Number of Comparisons					1		1			
Mean Effect Size					-0.88 ns		0.84 ns			
**ECT 24 h**										
Mean Baseline Value		69.65								8.64
Mean Percent Change		6.2%								34.3%
Number of Comparisons		1								1
Mean Effect Size		0.51 ns								0.90 ns

All treatments on this table are represented by only one study except ECT where the two studies employed different recording times.

ns indicates that original study or our reanalysis showed the change as non-significant, * indicates that the original study reported that the change was significant (*p *< .05).

**Table 3:** Effect Sizes for all measures in all studies examined.

**First Author (reference)**	**N**	**Design**	**Recording Time**	**Drug**	**Treatment Period (Weeks)**	*CB	**Effect Size (Cohen's d)**									
							**HR**	**SDNN**	**SDANN**	**RMSSD**	**LF**	**ln HF**	**HF**	**LF/HF**	**CV**	**pNN50**
	**TCAs Short time recording**															
Rechlin[55]	24	pre post	5 min	amitriptyline	2	Y	4.43	-1.61		-2.97			-0.87		-9.44	
Rechlin [56]	24	pre post	5 min	amitriptyline	2	Y	5.52	-2.59		-2.93			-1.19		-8.85	
Rechlin [57]	104	drug vs control	5 min	amitriptyline	1	Y	1.60	-1.53		-1.87			-0.54		-4.76	
Rechlin [58]	26	drug vs control	5 min	amitriptyline	2	Y	3.81	-2.55		-3.58			-1.25		-3.97	
Rechlin [59]	8	pre post	5 min	amitriptyline	2	Y	6.11	-1.88		-3.66					-10.86	
Rechlin [59]	8	pre post	5 min	doxepin	2	Y	7.87	-3.51		-5.04					-21.44	
Tulen[9]	9	pre post	8 min	imipramine	4	Y	4.81	-2.44				-6.43			-3.97	
Volkers[60]	24	pre post	5 min	imipramine	4	Y	4.52	-3.32				-2.64			-5.52	
	**TCAs Long time recording**															
Khaykin[51]	9	pre post	24 h	doxepin	6	N		1.80	3.64	-2.71						-3.34
Lederbogen[50]	14	pre post	24 h	amitriptyline	5	N	1.98	-2.20	-3.01							
	**SSRIs Short time recording**															
Rechlin [56]	24	pre post	5 min	paroxetine	2	Y	-0.45	0.05		-0.61			-0.30		-0.09	
Rechlin [59]	8	pre post	5 min	fluvoxamine	2	Y	-0.83	0.03		1.47					-0.42	
Rechlin [59]	8	pre post	5 min	paroxetine	2	Y	-0.77	0.09		-0.39					-0.27	
Straneva-Meuse[61]	17	vs control	2 min	paroxetine	8	N				-1.99						
Volkers[60]	17	pre post	5 min	fluvoxamine	4	Y	-0.47					-0.15				
	**SSRIs Long time recording**															
Khaykin[51]	5	pre post	24 h	fluoxetine	6	N		2.24	2.81	1.76						1.76
Lederbogen[50]	14	pre post	24 h	paroxetine	5	N	-0.10	-1.21	-1.95							
	**Other Treatments**															
Agelink[62]	25	pre-post	5 min	reboxetine	3	Y	1.32			-0.04	-0.38		0.79			
Agelink[63]	25	pre-post	5 min	nefazodone	3	Y	-1.60	0.08		0.11	-0.08		-0.20	-0.16	-0.21	
Straneva-Meuse[61]	17	vs control	2 min	bupropion	8	N				-1.99						
Tulen[9]	8	pre post	8 min	mirtazapine	4	Y	4.81	-2.44				-1.81			-3.97	
Karpyak[52]	11	pre post	24 h	ECT	**	NS		0.51								0.90
Nahshoni[53]	10	pre-post	NS	ECT	***	NS					-0.88		0.84			

HR = heart rate.

CV = coefficient of variability.

NS = Not Stated.

*CB = Controlled Breathing;.

**ECT performed 3 times per week to a maximum of 10 treatments.

***ECT performed 2 times per week to a maximum of 12 treatments.

## Discussion

The literature relating HRV to pharmacologic and physical treatments for depression is small. In the studies that do exist, there is considerable diversity in combinations of HRV measures used, as well as a lack of an obvious consistency in the manner in which measures were selected for a particular study. In our meta-analysis of the available literature, the short-term studies generated the most consistent results. With the short-term recording studies, TCAs (imipramine, doxepin, and amitriptyline) given to patients suffering from MDD were associated with a very large decrease in most measures of HRV and a large increase in HR, whereas the effect of SSRIs (fluvoxamine, fluoxetine and paroxetine) was much less clear. With SSRIs, we did find a significant decrease in heart rate and a marginally significant increase in only one measure of HRV, SDNN. These changes were of a much smaller magnitude compared to those associated with TCAs. Bupropion and mirtazapine also were associated with decreases in HRV. Clinically this indicates that SSRIs have less impact on heart rate and its variability than do TCAs and other drugs with anticholinergic effects, but that they do still reduce HR and increase SDNN.

There were only two studies using long-term recording of HRV, and these studies (which both looked at TCAs and SSRIs separately) generated inconsistent results. No systematic effect was observed for either class of drug in these two studies, and it is not possible to reconcile the differences between them as they examined different drugs in different populations (inpatients and outpatients).

There may be an explanation for our findings of less reliable effects with increased recording time. In recordings under controlled conditions (the short recordings), HRV is primarily vagally modulated, as well as being influenced by humoral factors, rhythmicity of the intrinsic cardiac pacemaker, and the effects of respiration. In 24 hour recordings the HRV is also influenced by activity and environmental factors. The inconsistent findings of the long recording studies may be due in part to the effects of the drugs on physical activity. Because patients do not remain supine during the recording period, changes in physical activity over 24 hours would lead to changes in HRV not directly related to the physiological effects of the drug (and in the case of TCAs in the opposite direction to those of the drug), and these changes would tend to obscure the physiological effects of the drug. This idea is supported by the fact that Khaykin [51] and Karpyak [52], both reported increases in some measures of HRV in individuals who responded to treatment compared to those who did not respond. Perhaps the changes in physical activity in those who responded to treatment that led to the increased HRV observed in these 24 hour studies for responders relative to non-responders decreased the reliability of the measures.

The consensus among the authors of the various studies is that increases in HR and decreases in HRV are due, at least in part, to the anticholinergic (i.e., antimuscarinic) properties of TCAs and of bupropion and mirtazapine, leading to diminished cardiac vagal tone.

Nahshoni and colleagues [53] conclude that the literature has not shown a reduction in HRV associated with SSRIs, and they suggest that SSRIs may in fact *increase *vagal modulation. The present data partially support this conclusion. We found a small increase in SDNN with SSRIs but not on other measures of HRV. The studies that we reviewed examining HRV and the treatment of depression have generally employed small samples of depressed, but otherwise medically well individuals, and the only reliable result in our review is that there is a possibly an increase in SDNN recorded while the participant was supine, with drugs in this class. The fact that the other measures do not show such a change is not in any way conclusive, as the individual studies report a wide range of changes in measures of HRV ranging from a reduction of 27% to an increase of 20%. This finding suggests that methodological factors may be influencing the results. However, our failure to find reliable changes in HRV with SSRIs is consistent with data from a large sample of cardiac patients as reported in the SADHART study [54]. That study failed to show significant changes in several measures of HRV with sertraline in depressed individuals who were not medically well. Further research is required to clarify whether the causes of variability between studies of SSRIs is due to differences in measurement or difference in medications.

## Conclusion

This review highlighted several important difficulties associated with research in the area of HRV. First, there exists a paucity of studies examining the effects of antidepressants on HRV; second, with one exception, published studies have used very small sample sizes, ranging from n = 6 to 24 (SSRIs), and n = 8 to 104 (TCAs); third, a variety of methodologies were used in assessing and measuring HRV; fourth, recording times vary between studies; and fifth, not all studies use the same medication within medication groups. The present study confirms that three TCAs decrease HRV and increase HR. The findings for the three SSRIs are less clear; however they do indicate a decrease in HR and a possible increase in SDNN with SSRI treatment. Increased HRV with SSRIs may have, theoretically at least, positive outcome implications with regard to morbidity and mortality. The less than robust effect that has been demonstrated may to some extent explain why only a statistical trend to better outcomes in depressed, recent post-MI patients receiving a SSRI was observed in the SADHART study. Further studies of the effect of specific SSRIs on HRV using consistent methodology are required.

## List of Abbreviations

HRV: heart rate variability; HR: Heart rate; NN: Normal to normal; SDNN: Standard deviation of all NN intervals; SDANN: Standard deviation of the 5-minute average NN intervals; RMSSD: Square root of the mean of the squared differences between adjacent NN intervals; CV: Coefficient of variation; HF: High frequency; LnHF: og transformation of HF. LF: Low frequency; VLF: Very low frequency; PNN50: the percentage of NN interval differences >50 ms from the preceding interval.

## Competing interests

The study was unfunded. The lack of funding did not limit the ability of the authors to research and produce this paper. No conflicts of interest were identified. None are declared.

## Authors' contributions

Concept and design: LTVZ, TH. Acquisition of data: LTVZ, TH. Analysis and interpretation of data: LTVZ, TH. Drafting of manuscript: LTVZ, TH. Critical revision of manuscript: KN.
